# A Streamline Strategy for Indication and Length of Telemetry Monitoring After TAVR

**DOI:** 10.1002/ccd.70299

**Published:** 2025-11-05

**Authors:** Antonin Fournier, Pierre Robert, Benoit Lattuca, Claire Duflos, Maël‐Morvan Duroyon, Jean‐Christophe Macia, Laurent Schmutz, Matthieu Steinecker, Jean‐Michel Berdeu, Thomas Gandet, Jean Luc Pasquie, François Roubille, Guillaume Cayla, Florence Leclercq

**Affiliations:** ^1^ Department of Cardiology CHU Montpellier Montpellier France; ^2^ Department of Cardiology CHU Nîmes Nîmes France; ^3^ Clinical Research and Epidemiology Unit CHU Montpellier, Univ Montpellier Montpellier France; ^4^ Department of Cardiac Surgery CHU Montpellier Montpellier France

**Keywords:** conductive disorder (CD), ECG telemetry monitoring (TM), intensive care, length of stay, pacemaker, transcatheter aortic valve replacement (TAVR)

## Abstract

**Background:**

Both indication and length of ECG telemetry monitoring (TM) after transcatheter aortic valve replacement (TAVR) are not well defined.

**Aims:**

We hypothesize that a targeted strategy for monitoring conductive disorders (CD) post‐TAVR can reduce the need and duration of in‐hospital TM without compromising safety.

**Methods:**

We prospectively evaluated consecutive patients undergoing transfemoral TAVR between February 2023 and September 2024. Intensive care unit (ICU) transfer for TM was considered when previous right bundle branch block or when new or worsening CD. TM duration was standardized, from 24 to 48 h. The primary endpoint was incidence of severe CD, defined as symptomatic or requiring medical intervention, occurring outside the ICU, at 1‐month follow‐up.

**Results:**

Among 250 patients included (mean age: 80.53 ± 6.86 years), 138 (55.20%) required TM, mainly due to new left bundle branch block (*n* = 64, 44.80%). By respecting the protocol, the primary endpoint was achieved for three patients (1.20%, 95% CI: 0.31; 3.77), all related to post‐discharge CD occurring after Day 6 and requiring permanent pacemaker implantation (PPI). There were no deaths and no severe in‐hospital CD outside the ICU. The PPI rate was 16.80%. The mean ICU length of stay was 1.34 days. Absence of TM was associated with shorter mean global hospitalization duration (1.43 vs. 2.93 days, *p* < 0.001).

**Conclusion:**

Selective indication and length of TM after TAVR is possible with no in‐hospital events, no deaths, and a low rate of rhythmic events after hospital discharge, confirming the safety of this in‐hospital strategy. Nearly half of the patients did not require TM, and very short global and ICU lengths were observed.

**Abbreviations**
AEM: ambulatory ECG monitoring
AVB: atrioventricular block
CD: conductive disorder
CI: confidence interval
EPS: electrophysiology study
GCW: general cardiology ward
ICU: intensive care unit
IQR: interquartile range
LAH: left anterior block
LBBB: left bundle branch block
PPI: permanent pacemaker implantation
RBBB: right bundle branch block
SD: standard deviation
TAVR: transcatheter aortic valve replacement
THV: transcatheter heart valve
TM: ECG telemetry monitoring
TU: telemetry unit

## 1 Introduction

Transcatheter aortic valve replacement (TAVR) has become a standard of care for patients with symptomatic aortic stenosis and is currently associated with a low rate of adverse events [1, 2]. However, high degree conductive disorders (CD) requiring permanent pacemaker implantation (PPI) have modestly decreased over time and remain the main complications of the procedure [3, 4]. New‐onset left bundle branch block (LBBB) is the leading reason for monitoring patients in intensive care unit (ICU) or in telemetry unit (TU) following TAVR [5]. Both indication and duration of ECG telemetry to monitor CD after TAVR are, however, not well defined, more based on expert consensus than on specific studies [6, 7, 8]. Results from the 2022 European Heart Rhythm Association (EHRA) highlight important heterogeneity in the management of CD and risk stratification strategies after TAVR across European centers [9]. Previous studies suggest that the risk of in‐hospital severe CD post‐TAVR may be anticipated when patients were carefully selected regarding ECG analysis pre‐ and postprocedure [10, 11, 12]. However, despite the development of fast‐track protocols, most patients undergo empiric 12−24 h ECG telemetry monitoring (TM) with or without ICU admission after TAVR, even though the majority will not experience any rhythmic event [6, 7, 9, 10]. Furthermore, we lack data regarding the optimal duration of TM post‐TAVR when CD occurred. An in‐hospital algorithm for screening and monitoring CD after TAVR could rationalize both indication and duration of TM monitoring as well as need for PPI and help to early hospital discharge. Our aim is to reduce the indications and duration of ECG monitoring (with telemetry or in ICU) after TAVR, to decrease the overall length of hospitalization without compromising patient safety. We hypothesize that no severe CD will occur during hospital stay in patients without TM [10, 11, 12], with a low rate of severe CD and no rhythmic death after discharge at 1‐month follow‐up. We anticipate that 15% of patients monitored will develop a severe CD requiring PPI [3, 4].

## 2 Methods

### 2.1 Study Design

We designed a non‐comparative, prospective interventional study with the main objective to evaluate the effectiveness and safety of an in‐hospital strategy for screening, monitoring, and managing CD after TAVR with the aim to prevent the occurrence of severe CD occurring outside the ICU during in‐hospital stay and at 1‐month follow‐up. Inclusion lasted from February 2023 to September 2024.

The study protocol was approved by an independent ethics committee (Comité de Protection des Personnes Sud Méditerranée, Montpellier, France (CPP MTP n°22.01444.0000) and all patients provided oral consent. No additional testing or biological samples were specifically required for the study.

The trial was conducted in accordance with the World Medical Association Declaration of Helsinki and was registered with ClinicalTrials.gov under the identifier NCT 05417464.

### 2.2 Patients

The Heart Team established the indication for TAVR according to current guidelines [2].

All consecutive patients who underwent transfemoral TAVR at our center (Montpellier University Hospital, France) during the study period were considered to be included in the study. Patients with prior pacemaker or those requiring ICU monitoring for other reasons than CD, such as vascular complication, hemodynamic instability, or LVEF < 30%, were excluded from the study.

Details on ECG data regarding the definition of CD are usual and described in Supporting Information S1: Data 1. We considered, in particular, previous right bundle branch block (RBBB), extreme bradycardia of any kind ( < 40 bpm), atrioventricular block (AVB) of any degree, and LBBB.

### 2.3 Procedural Aspects

All patients underwent TAVR procedures using the latest generation devices, including balloon‐expandable Sapien 3 or Sapien 3 Ultra (Edwards Lifesciences, Irvine, California), self‐expandable Evolut R, PRO, or PRO+ (Medtronic, Minneapolis, Minnesota), or Navitor (Abbott, Chicago). TAVR procedures were usually performed under local anesthesia with sedation. Transfemoral access was performed with echography guidance and using percutaneous closure (mainly two Proglides, Abbott, Chicago). Pre‐dilatation of the valve was not the rule and was left at the operator's discretion. A final control was performed by aortography and transthoracic echocardiography. Procedural success was defined as the deployment of a single valve with a mean gradient < 20 mmHg and an aortic regurgitation grade < 2 according to the VARC‐3 criteria [13].

All patients underwent TM for 1 h in the anesthesia recovery room followed by a 12‐lead ECG before deciding the transfer to the ICU or the general cardiology ward (GCW).

ICU provided 24/7 continuous TM, hemodynamic support, and noninvasive ventilation assistance, whereas the GCW was a standard cardiology unit, not allowing TM.

### 2.4 Management of CD

Patients were assessed for the risk of CD using a specific protocol involving 12‐lead ECG analysis performed pre‐procedure and 1 h after TAVR (Central Illustration illustration 1, 1). The algorithm was based on previous findings regarding the evolution and risk of CD after TAVR [10, 11, 14].

**Central illustration 1:**
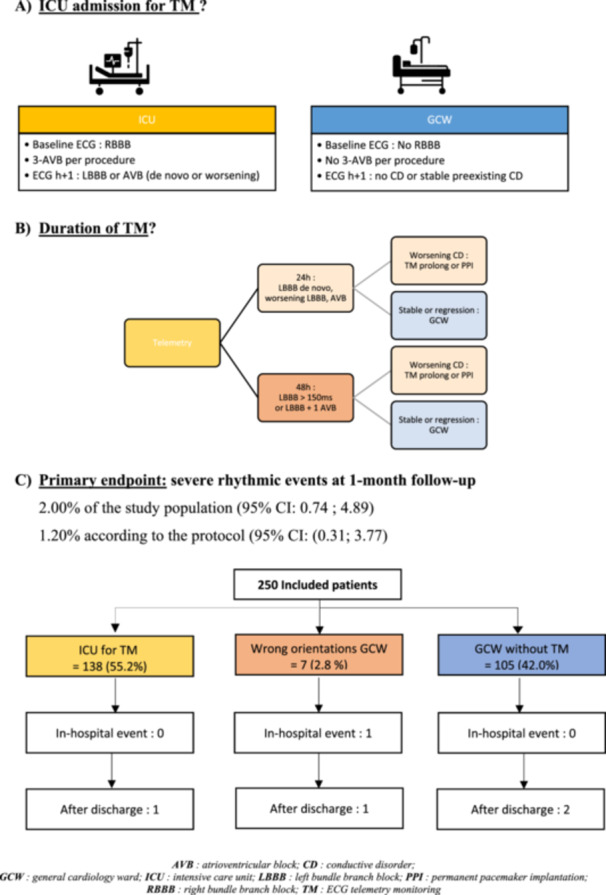
Algorithm of management of conductive disorders after TAVR. (A) ICU admission for TM? (B) Duration of TM? (C) Primary endpoint: severe rhythmic events at 1‐month follow‐up. 2.00% of the study population (95% CI: 0.74; 4.89). 1.20% according to the protocol (95% CI: (0.31; 3.77). AVB, atrioventricular block; CD, conductive disorder; GCW, general cardiology ward; ICU, intensive care unit; LBBB, left bundle branch block; PPI, permanent pacemaker implantation; RBBB, right bundle branch block; TM, ECG telemetry monitoring. [Color figure can be viewed at wileyonlinelibrary.com]

Patients considered “at risk” for high degree CD included those with pre‐existing RBBB, new onset or worsening LBBB > 20 ms, new AVB of any degree, or a ventricular rate < 40 bpm [6, 7, 9, 10]. These patients were admitted to the ICU for TM.

Patients considered “not at risk” (all other patients) were admitted to the GCW without TM unless they had other non‐rhythmic indications requiring specific monitoring or ICU transfer, in which case they were excluded.

The duration of TM was based on the type and evolution of CD, as stipulated in the algorithm: 24 h for pre‐existing RBBB; 24 h for new onset LBBB; 48 h for LBBB associated with first‐degree AVB (1‐AVB) or wide LBBB > 150 ms (Supporting Information S1: Data 2).

Transfer to the GCW was allowed after the planned ICU stay if no new or worsening CD was detected. A temporary pacemaker was placed only in cases of 3‐AVB with a wide QRS or no escape rhythm.

PPI was indicated following TM in cases of RBBB combined with any other conduction abnormality or when CD worsened during TM (Central Illustration illustration 1, 1, Supporting Information S1: Data 2).

Ambulatory ECG monitoring (AEM) with Holter or intracardiac monitoring (ICM) was recommended at discharge in selected high‐risk patients without PPI indication according to our algorithm. Details on CD management are provided in Supporting Information S1: Data 2.

### 2.5 Study Endpoints

The primary endpoint was a safety endpoint, defined as the rate of patients with severe CD including those symptomatic (associated with syncope, sudden death, or heart failure) or those requiring specific management (drug treatment, prolonged hospitalization, need for transient pacing, or PPI) and occurring outside the ICU, whether or not the patient was initially admitted in this unit, at 1‐month follow‐up.

The secondary endpoints included the incidence and type of CD requiring ICU transfer for TM, PPI rate and indications, time of onset of CD requiring PPI, evolution of CD not considered to require PPI, length of hospital stay in ICU for TM, and in the GCW.

Clinical outcomes were collected during hospitalization and at 1‐month follow‐up. Patient follow‐up included an intra‐hospital visit and a 1‐month post‐TAVR phone call to assess primary and secondary outcomes and clinical status.

### 2.6 Statistical Analysis

We expect that, by using our algorithm, the incidence of severe CD will be almost non‐existent outside the ICU during the hospital stay, and less than 5% at follow‐up [15]. To ensure the acceptability of this management approach, it will be necessary to demonstrate that the incidence of in‐hospital severe CD outside the ICU remains below 1%. With a population of 250 patients, the one‐sided 95% confidence interval (CI) will not exceed 1% if the actual incidence is less than 0.3% (NQuery calculation using the POC6 procedure with the simple asymptotic method).

Population characteristics, in‐hospital evolution, and treatment were described in patients with or without TM. Baseline characteristics were presented as mean ± standard deviation (SD) or median and interquartile range (IQR) depending on the distribution of the variable for continuous variables, and frequencies and proportions for categorical variables. The primary analysis was the incidence rate of severe CD, excluding the ICU stay period. We reported the incidence rate with its CI. Independent non‐parametric group comparison tests were performed to assess the distribution of bioprosthesis models selected for the TAVR procedures and the duration of ICU and GCW stays.

Statistical analysis was performed using R software version 4.3.1 (R Foundation for Statistical Computing). A two‐sided *α* value of < 0.05 was required for statistical significance for all tests.

## 3 Results

### 3.1 Patients' Characteristics

A total of 303 consecutive patients underwent femoral TAVR during the study period. Among these, 250 patients were included in the final analysis (Flowchart, Figure illustration 1, 1).

**Figure 1:**
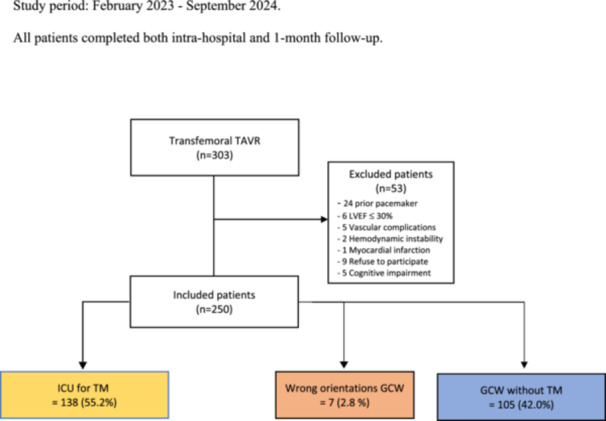
Flowchart. Study period: February 2023 to September 2024. All patients completed both intra‐hospital and 1‐month follow‐up. [Color figure can be viewed at wileyonlinelibrary.com]

The main baseline characteristics of the study population were similar between the two groups except for ECG and are summarized in Tables 1 and 2. The mean age was 80.53 ( ± 6.86) years, and 52.80% of patients were male. The mean Euroscore II was 3.25 ( ± 3.65) and the mean STS score 2.46 ( ± 2.61). Baseline ECG findings included atrial fibrillation in 16.40% of patients, 1st degree AVB (1‐AVB) in 23.60%, LBBB in 13.20%, and RBBB in 11.20%. Prevalence of RBBB was higher and QRS duration was longer in the ICU group (Table 2).

**Table 1:** Baseline characteristics of patients: total population and according to ICU or GCW transfer.

Variable	Population	A–GCW (*n* = 112)	B–ICU (*n* = 138)
Age (years), mean ( ± SD)	80.53 ( ± 6.86)	80.21 ( ± 6.96)	80.79 ( ± 6.78)
Sex (male), *n* (%)	132 (52.80)	58 (51.79)	74 (53.62)
BMI (kg/cm²), mean ( ± SD)	27.29 ( ± 5.50)	27.08 ( ± 5.91)	27.46 ( ± 5.17)
NYHA, *n* (%)			
I or II	186 (74.40)	83 (74.11)	103 (74.64)
III or IV	64 (25.60)	29 (25.89)	35 (25.36)
STS score, mean ( ± SD)	2.46 ( ± 2.61)	2.34 ( ± 1.88)	2.55 ( ± 3.08)
EuroSCORE 2, mean ( ± SD)	3.25 ( ± 3.65)	2.87 ( ± 1.90)	3.56 ( ± 4.60)
Hypertension, *n* (%)	189 (75.60)	82 (73.21)	107 (77.54)
Diabetes, *n* (%)	85 (34.00)	31 (27.68)	54 (39.13)
Dyslipidemia, *n* (%)	145 (58.00)	62 (55.36)	83 (60.14)
Smoking, *n* (%)	66 (26.40)	30 (26.79)	36 (26.09)
History of atrial fibrillation or flutter, *n* (%)	57 (22.80)	29 (25.89)	28 (20.29)
Heart failure within 12 months, *n* (%)	22 (8.80)	8 (7.14)	14 (10.14)
Stroke, *n* (%)	25 (10.00)	7 (6.25)	18 (13.04)
Ischemic heart disease, *n* (%)			
Coronary bypass	9 (3.60)	2 (1.79)	7 (5.07)
Angioplasty	46 (18.40)	22 (19.64)	24 (17.39)
Medical therapy	9 (3.60)	5 (4.46)	4 (2.90)
No	186 (74.40)	83 (74.11)	103 (74.64)
Peripheral arterial disease, *n* (%)			
Lower limb	10 (4.00)	2 (1.78)	8 (5.80)
Carotid stenosis/endarterectomy	20 (8.00)	7 (6.25)	13 (9.42)
No	220 (88.00)	103 (91.96)	117 (84.78)
Valve surgery history, *n* (%)			
Aortic	5 (2)	3 (2.68)	2 (1.44)
Mitral	1 (0.4)	0 (0.00)	1 (0.72)
No	244 (97.60)	109 (97.32)	135(97.82)
GFR (CKD‐EPI), mean ( ± SD)	58.26 ( ± 21.66)	59.45 ( ± 22.12)	57.28 ( ± 21.30)
CRP (mg/L), mean ( ± SD)	6.21 ( ± 11.95)	6.55 ( ± 13.87)	5.94 ( ± 10.20)
Troponin T‐hs (ng/L), mean ( ± SD)	52.96 ( ± 245.02)	39.12 ( ± 77.08)	64.28 ( ± 323.10)
Hemoglobin (g/dL), mean ( ± SD)	14.38 ( ± 15.14)	13.43 ( ± 10.80)	15.15 ( ± 17.91)
Platelets (g/L), mean ( ± SD)	215.93 ( ± 67.79)	210.94 ( ± 68.26)	219.93 ( ± 67.39)
LVEF (%), mean ( ± SD)	57.99 ( ± 8.56)	58.19 ( ± 8.60)	57.83 ( ± 8.55)
Aortic gradient (mmHg), mean ( ± SD)	48.51 ( ± 11.14)	47.69 ( ± 11.28)	49.18 ( ± 11.02)
Aortic valve area (cm²), mean ( ± SD)	0.79 ( ± 0.19)	0.78 ( ± 0.19)	0.80 ( ± 0.19)
Betablocker, *n* (%)	96 (38.40)	47 (41.96)	49 (35.51)
Non‐dihydropyridine calcium channel blocker, *n* (%)	7 (2.80)	3 (2.68)	4 (2.90)
Amiodarone, *n* (%)	20 (8.00)	10 (8.93)	10 (7.25)

**Table 2:** ECG characteristics of patients: total population and according to ICU or GCW transfer.

Variable	Population	A–GCW (*n* = 112)	B–ICU (*n* = 138)	*p* value
Heart rate (bpm), mean ( ± SD)	71.29 ( ± 12.49)	70.34 ( ± 12.35)	72.06 ( ± 12.59)	0.121^a^
Atrial fibrillation or flutter, *n* (%)	41 (16.40)	17 (15.18)	24 (17.39)	0.661^b^
PR interval, mean ( ± SD)	186.20 ( ± 37.06)	182.19 ( ± 33.74)	189.54 ( ± 39.44)	0.289^a^
1‐AVB, *n* (%)	59 (23.60)	25 (22.32)	34 (24.64)	0.576^b^
QRS duration (ms), mean ( ± SD)	59 (23.60)	25 (22.32)	34 (24.64)	**0.016** a
Bundle branch block, *n* (%)				**0.002** ^b^
Incomplete RBBB ( < 120 ms)	0 (0.00)	0 (0.00)	0 (0.00)	
Complete RBBB ( > 120 ms)	28 (11.20)	1 (0.89)	27 (19.56)	
Incomplete LBBB ( < 120 ms)	19 (7.60)	9 (8.04)	10 (7.24)	
Complete LBBB ( > 120 ms)	14 (5.60)	6 (5.36)	8 (5.80)	
None	189 (75.60)	96 (85.71)	93 (67.40)	
Hemiblock, *n* (%)				0.520^b^
Left anterior hemiblock (< −30°)	34 (13.60)	14 (12.50)	20 (14.49)	
Left posterior hemiblock ( > 90°)	0 (0.00)	0 (0.00)	0 (0.00)	
None	216 (86.40)	98 (87.50)	118 (85.51)	

^a^ Wilcoxon rank sum test.

^b^ Pearson's chi‐squared test.

### 3.2 Procedural Details

TAVR procedures were performed under local anesthesia with sedation in 84.80% of cases. Balloon expandable devices were mainly used (76.00% of patients). Procedural success was achieved in 247 of the patients (98.80%). In three patients (1.20%), two prostheses were required.

### 3.3 TM Strategy (Figure 2)

**Figure 2:**
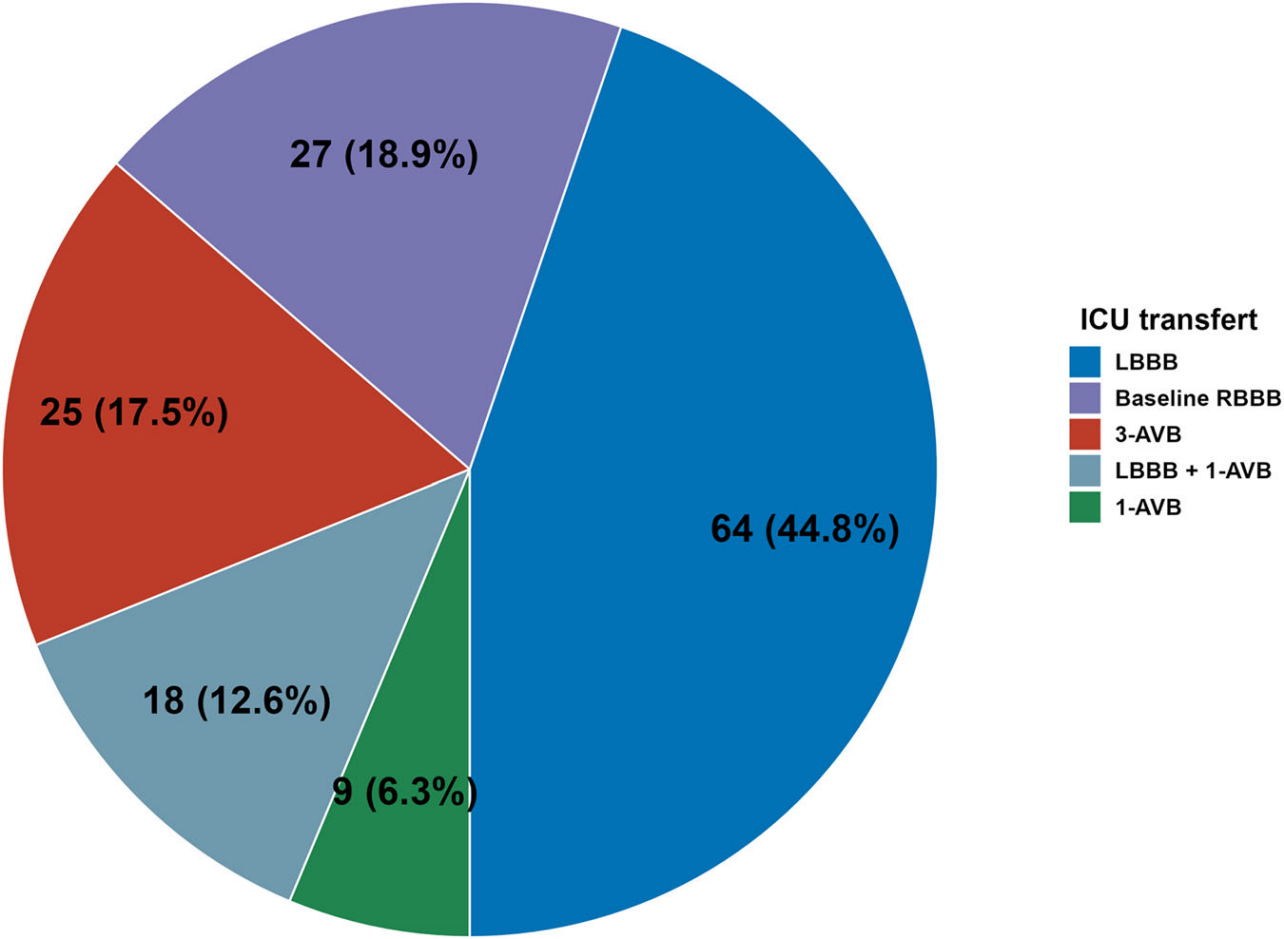
Indications of ICU transfer for telemetry monitoring (*n* = 143). [Color figure can be viewed at wileyonlinelibrary.com]

A total of 27 patients were directly transferred to the ICU for TM regardless of the procedural course due to pre‐existing RBBB. After 1 h of monitoring in the anesthesia recovery room, an additional 111 patients were transferred to the ICU for TM. New onset or worsening LBBB (64 patients) was the most common reason for TM. Overall, 55.20% of patients were transferred to the ICU for TM 1 h after the procedure.

Patients with noncompliance with the protocol (*n* = 8) are described in Supporting Information S1: Data 3.

### 3.4 Primary Endpoint

The primary endpoint occurred in five patients, representing 2.00% of the population (95% CI: 0.74; 4.89) (Central Illustration illustration 1, 1). All these patients have received a Sapien 3 Ultra transcatheter heart valve (THV).

Among the five patients, one was incorrectly assigned to the GCW despite having a wide LBBB associated with a new 1‐AVB, and experienced syncope on Day 3 leading to PPI. Additionally, one other patient did not receive PPI despite indication (pre‐existing RBBB and new 1‐AVB) and developed 3‐AVB on Day 6, leading to PPI (Supporting Information S1: Data 3).

By respecting the protocol, three patients experienced nonfatal CD events representing 1.20% of the study population (95% CI: 0.31; 3.77), all occurring after hospital discharge (between Days 6 and 8) and requiring PPI.

The first patient, who had previous left anterior block (LAH), developed a 1‐AVB after TAVR and was discharged on Day 3. He experienced a non‐traumatic syncope on Day 6 related to 3‐AVB.

The second patient developed a new onset of wide LBBB and was fitted with an ICM. He was discharged on Day 6 and experienced a non‐traumatic syncope on Day 7 related to 3‐AVB.

The third patient had no CD at discharge on Day 3. He experienced dizziness on Day 8; the ECG revealed a new onset of LBBB with 1‐AVB.

ECGs of these three patients are reported in Supporting Information S1: Data 4.

Notably, no severe rhythmic events occurred during the in‐hospital stay, and there were no deaths at 1‐month follow‐up.

### 3.5 Secondary Endpoints

**Figure 3:**
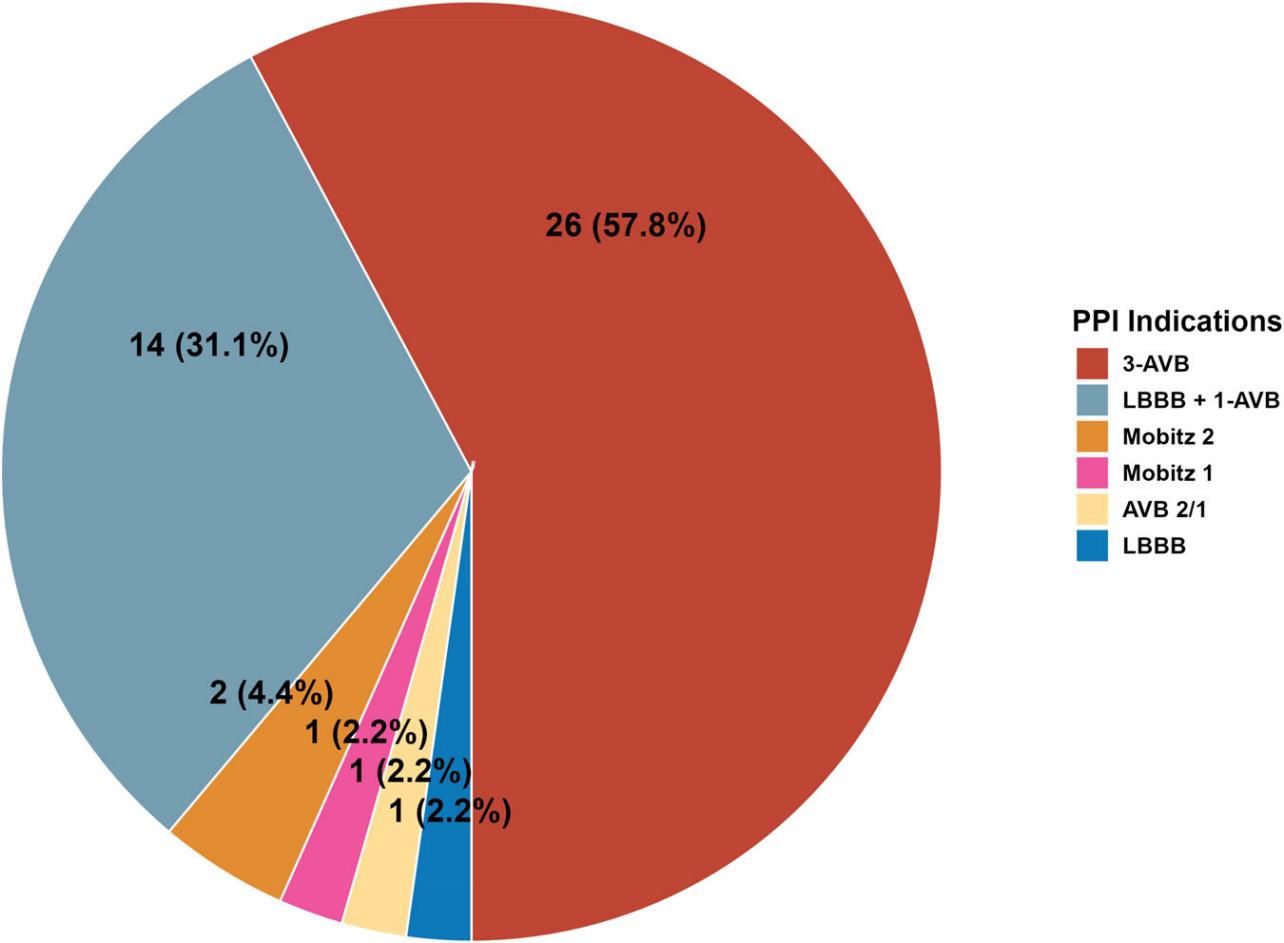
Permanent pacemaker implantation indications (*n* = 45). [Color figure can be viewed at wileyonlinelibrary.com]

During the hospital phase, new‐onset LBBB occurred in 98 patients (39.20%) with 15 of them (15.15%) requiring PPI. 3‐AVB occurred in 32 patients (12.80%), with 24 of them (75.00%) requiring PPI. The primary indication for PPI was 3‐AVB (57.78%), followed by LBBB associated with 1‐AVB (31.11%) (Figure 3).

**Figure 4:**
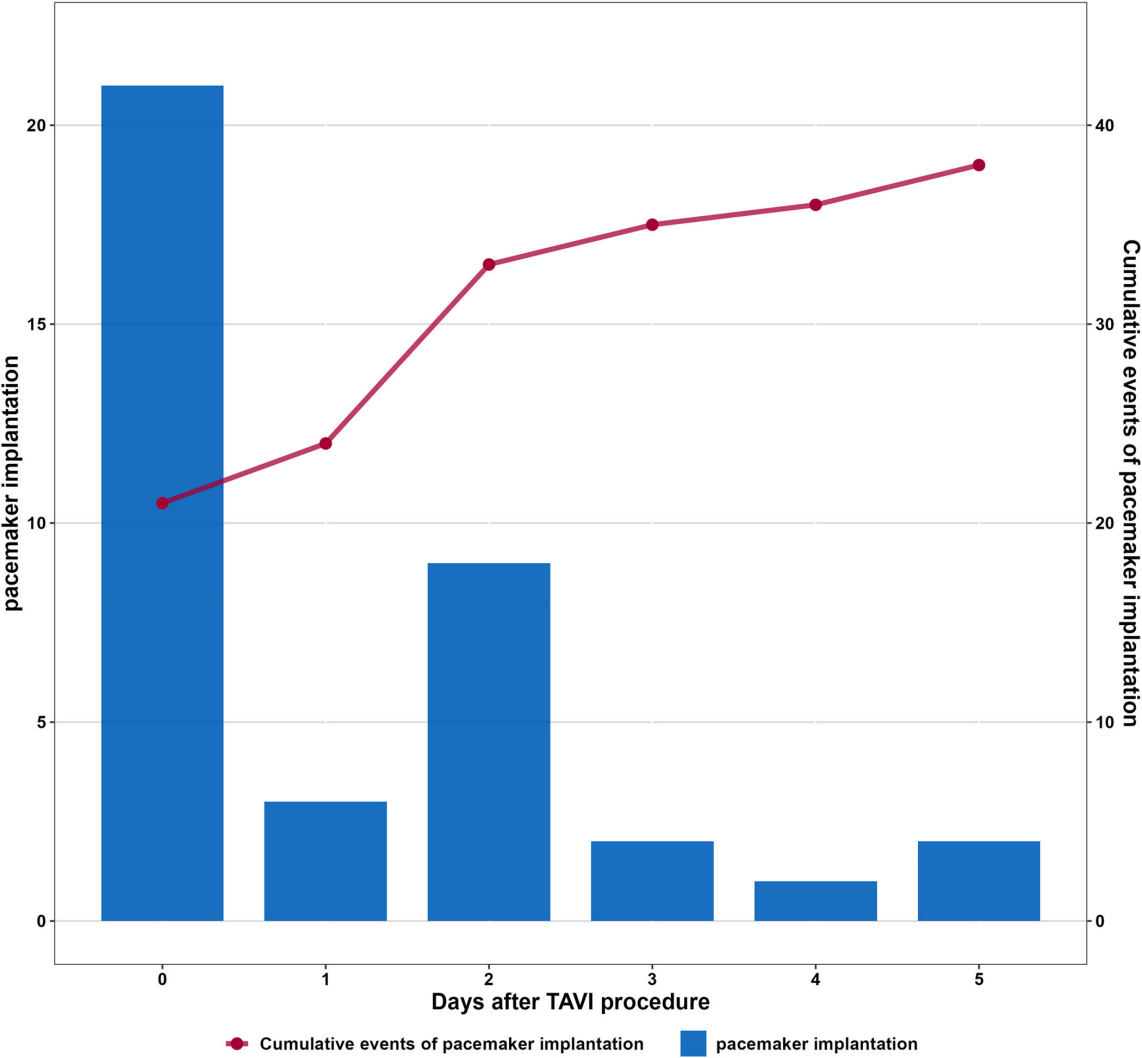
Timing of in‐hospital permanent pacemaker implantation. [Color figure can be viewed at wileyonlinelibrary.com]

During the hospital stay, 38 patients (15.20%) required PPI. At 1‐month follow‐up, the PPI rate had increased to 16.80% (*n* = 42). The majority of PPI (*n* = 33, 78.57%) was performed within the first 48 h postprocedure, with an additional 11.90% during hospital stay and 9.50% after discharge (Figure 4). We did not observe a difference regarding the type of THV and incidence of PPI (Supporting Information S1: Data 5).

CD not requiring PPI occurred in 102 patients, primarily due to new‐onset LBBB (*n* = 83), isolated 1‐AVB (*n* = 20), or transient periprocedural 3‐AVB (*n* = 8).

The mean hospitalization durations are presented in Table 3 and were significantly shorter for patients without ICU admission (*p* < 0.001). The mean length of ICU stay was 1.34 days ( ± 0.74). The mean total hospitalization length of stay was 2.30 ( ± 2.19) days.

**Table 3:** Length of stay (ICU, GCW, total) in patients with or without initial ICU admission.

Variable	Population	A—GCW (*n* = 112)	B—ICU (*n* = 138)	*p* value
Total stay (days)				**< 0.001** a
Mean ( ± SD)	2.30 ( ± 2.19)	1.43 ( ± 1.14)	2.93 ( ± 2.54)	
Median (IQR)	1.00 (1.00; 3.00)	1.00 (1.00; 1.75)	2.00 (1.00; 4.00)	
ICU stay (days)				
Mean ( ± SD)	1.34 ( ± 0.74)	/	1.34 ( ± 0.74)	
Median (IQR)	1.00 (1.00; 2.00)	/	1.00 (1.00; 2.00)	
GCW stay (days)				**< 0.001** a
Mean ( ± SD)	1.99 ( ± 1.99)	1.42 ( ± 1.12)	2.73 ( ± 2.57)	
Median (IQR)	1.00 (1.00; 2.00)	1.00 (1.00; 1.75)	2.00 (1.00; 4.00)	

^a^ Wilcoxon rank sum test.

## 4 Discussion

While management strategies for CD occurring after TAVR remain controversial, this prospective study aimed to evaluate the efficacy and safety of an algorithm designed to rationalize both the indications and duration of TM after TAVR and to decrease the safety of hospital length of stay.

### 4.1 Several Key Findings Were Highlighted

1. Our study confirms the safety of a targeted strategy for TM indication and length after TAVR, with no deaths or severe rhythmic events occurring during hospitalization and only rare and nonfatal rhythmic events at 1‐month follow‐up (1.2%).
2. Nearly half of the patients did not require TM using this protocol.
3. The strategy was associated with very short hospitalization length of stay, particularly in patients who did not require TM.
4. Using this in‐hospital algorithm, delayed CD after hospital discharge were rare but possible.

### 4.2 Primary End Point: Safety of the Strategy

The safety of a selected TM strategy after TAVR, highlighted in our study, is reassuring. No deaths or serious rhythmic events occurred outside the ICU during hospitalization. These results confirm previous findings regarding the possibility of anticipating the risk of in‐hospital CD after TAVR when patients are carefully selected [10, 11, 12].

The incidence of serious rhythmic events occurring outside the ICU was 1.20% (*n* = 3), all observed after hospital discharge, beyond Day 6. These events occurred beyond the usual hospital length of stay for TAVR, supporting the safety of this in‐hospital strategy. This 1.20% incidence of serious rhythmic event at 1 month compared favorably with recent reports related to late CD after TAVR and argues to an appropriate selection of patients requiring PPI during hospital stay using our strategy [15].

Interestingly, two of these events occurred in patients with CD at discharge not considering as requiring PPI according to our algorithm and were non‐traumatic syncopes.

The first patient had previous LAH and a new but stable 1‐AVB at discharge. LAH has, however, proven to be a significant but moderate risk factor for PPI after TAVR. More caution may be warranted for these patients in future management strategies, particularly when associated with 1‐AVB [16, 17].

The second patient with new wide but stable LBBB was discharged with ICM while he was considered at risk for delayed high‐degree CD but without a definite indication for PPI [18].

The third event, which did not involve syncope, occurred in a patient without any CD at discharge. This observation highlights the need to better define the risk of delayed CD beyond the standard ECG analysis.

This study aims to limit subjectivity for PPI and to decrease the risk of under‐ or overtreatment.

Finally, this strategy resulted in a 1‐month PPI rate comparable to those reported in recent studies and registries [4, 9, 19].

### 4.3 TM After TAVR

With this strategy, nearly half of the patients did not require ICU transfer for TM and were eligible for early discharge, potentially improving patient comfort and procedural cost‐effectiveness. Recently developed fast track protocols generally tend toward a more conservative approach, with TM lasting 24 h or overnight for all patients after TAVR [6, 20].

A strategy of selective TM after TAVR may optimize resource utilization, reduce TU and ICU overload by allowing low‐risk patients to be admitted directly to the GCW without TM. This approach is particularly relevant in high‐volume TAVR centers [10, 14].

Our study confirmed that CD was the main indication for ICU admission after TAVR, with only 14 patients (5.6%) transferred to the ICU for other reasons [19]. We excluded patients without a rhythm‐related indication for ICU admission to limit selection bias in ICU admission decisions, which could influence event rates and distort the apparent effectiveness of the strategy.

### 4.4 Length of Stay After TAVR

This algorithm proposed an original strategy for determining ECG monitoring duration based on pre‐existing or evolving CD after TAVR. The absence of severe rhythmic events outside the ICU during the hospital stay supports the effectiveness and safety of this strategy, but could be confirmed in a multicenter and randomized study.

The observed short mean hospitalization length (2.30 days), particularly in patients without TM (1.43 days), has a potential economic impact, aligning with results of recent studies focused on optimizing length of stay and quality of care after TAVR [21, 22]. Moreover, extended hospitalization has been associated with an increased risk of complications and mortality in older patients undergoing TAVR [23].

### 4.5 Prediction of Late CD

Main late serious rhythmic events after TAVR typically occurred in patients with CD at discharge [11, 12]. However, patients with normal conduction at discharge can still experience late‐onset heart block [15, 24].

The utility of an electrophysiology study (EPS) remains debated and may be proposed in selected patients to help in CD risk stratification after TAVR [25].

Although not systematically performed in our study, evaluating anatomic factors such as membranous septum length by computer tomography could potentially improve risk stratification for postprocedural CD, whereas its value for predicting delayed CD remains to be demonstrated [26, 27].

In the context of early discharge after TAVR, AEM has emerged as a valuable tool for detecting delayed CD, especially in the first month after TAVR [28]. Current data support the use of AEM in selected patients (baseline RBBB, new‐onset LBBB, transient 3‐AVB) [7, 8].

Machine learning is increasingly leveraged to predict CD risk post‐TAVR, utilizing algorithms that analyze clinical, echographic, ECG, and CT imaging data, including membranous septum length. Recently, in an observational and mainly retrospective study, the D PACE score was proposed to identify low versus high‐risk patients regarding the occurrence of late CD [29]. The algorithm was interesting, with an incidence of CD in low‐risk patients similar to our strategy using only ECG criteria but excluded patients with atrial fibrillation. Furthermore, the authors did not evaluate the issue of the duration of TM, as we did in this study.

### 4.6 Study Limitations

This report consists of a single‐center observational study, and the limitations are firstly inherent to the study design, not allowing us to extend our results to other centers in settings with different levels of operator experience, patient demographics, or post‐TAVR management protocols. However, this algorithm is simple and could be assessed easily.

The relatively short follow‐up period may have underestimated the true incidence of CD, but in old patients, degenerative CD may occur later and may not necessarily be related to TAVR. Our aim was first to capture in‐hospital events to validate the safety of our in‐hospital strategy without monitoring. Delayed CD may be assessed by AEM or connected devices. While AEM could potentially improve post‐discharge safety, it was not proposed routinely in our study.

There was no centralized core laboratory for ECG analysis. However, all ECGs were reviewed by experienced cardiologists with automatic numeric analysis, which minimizes the risk of error.

Balloon‐expanding valves were predominantly used (76%) due to the operators' preferences at our center, and generally in Europe and the USA [30], which may introduce potential bias and did not allow for comparison of the risk of CD and the strategy between THV. Nonetheless, CD associated with the latest generation of all types of devices seems to be roughly comparable [4].

## 5 Conclusion

An in‐hospital strategy for selective indication and length of TM after TAVR evaluated in our study appears safe, with no deaths, no severe rhythmic events during hospital stay, and rare non‐traumatic rhythmic events occurring several days after discharge at 1‐month follow‐up. The proposed protocol allows to dispense of ICU or TM in nearly half of the patients after TAVR, with an optimized TM duration. This rationalized strategy results in very short ICU stay and global hospitalization duration, with potentially important medical and economic impacts.

While post‐discharge events may still occur, we need to improve the risk evaluation of delayed CD to select patients with an indication of AEM after discharge.

### 5.1 Highlights

1. The proposed strategy regarding indication and length of TM after TAVR is safe: no death, no severe CD outside the ICU during the hospital stay, rare and nonfatal rhythmic events at 1 month follow‐up (1.2%).
2. The strategy allows to avoid ICU in nearly half of the patients after TAVR and is associated with very short hospitalization durations.
3. Late out‐of‐hospital severe CD is rare but can still occur, even when the ECG at discharge is normal: impact of ambulatory or intracardiac ECG monitoring, evaluation of anatomic factors (membranous septum length), and the role of machine learning may be evaluated in future studies.

## Conflicts of Interest

The authors declare no conflicts of interest.

## Supporting information

Supp data CCI.
