# Supplementary material for: A Streamline Strategy for Indication and Length of Telemetry Monitoring After TAVR
Source: Catheter Cardiovasc Interv. 2025 Nov 5;107(1):142–52. doi: 10.1002/ccd.70299 (PMC12775202; doi:10.1002/ccd.70299)
Supplement: Supplementary file 1 — Supp data CCI. [file CCD-107-142-s001.docx]

**Supplementary data 1: ECG data and conductive disorders definitions**

*Electrocardiographic data* included a 12-led ECG obtained from all patients at baseline,
1h post procedure in the anesthesia recovery room, at admission to ICU or GCW, and daily until hospital discharge. Heart rate, PR interval, QRS duration and QRS axis, were determined at each ECG recording. All ECGs were analyzed by experienced cardiologists.

*Conductive disorders* were defined as follows:

- 1-AVB: PR interval > 200 ms.
- Complete LBBB: QRS duration > 120 ms and typical pattern of LBBB.
- Complete RBBB: QRS duration > 120 ms and typical pattern of RBBB.
- Incomplete LBBB: QRS duration between 80 – 120 ms and typical pattern of LBBB.
- Incomplete RBBB: QRS duration between 80 – 120 ms and typical pattern of RBBB.
- Left anterior hemiblock (LAH): left axis deviation < – 30°.
- Left posterior hemiblock (LPH): right axis deviation > 90°.
- High degree AVB: third-degree AV or second-degree type II (Mobitz II) heart block.

**Supplementary data 2: Detailed algorithm**

**
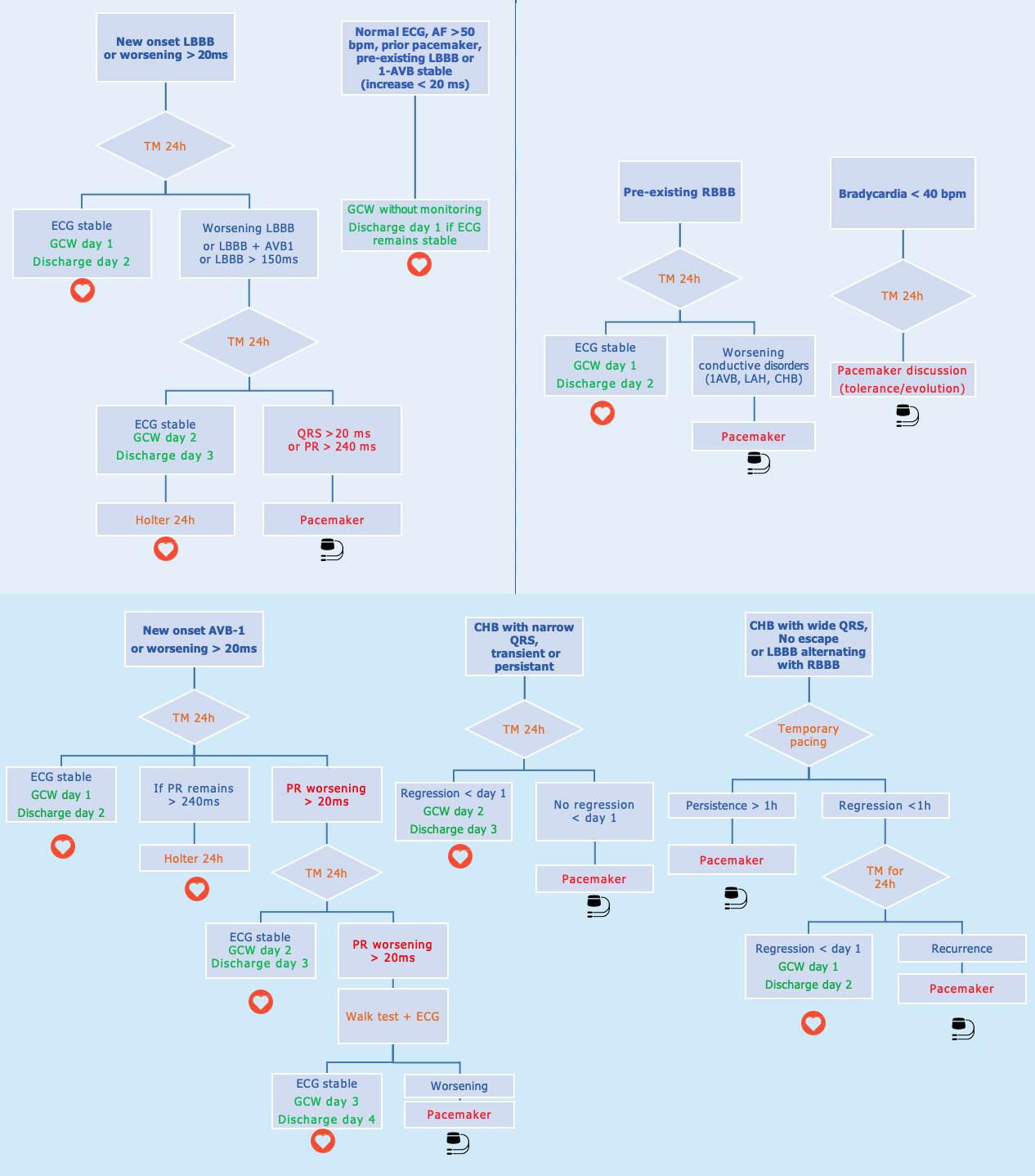
**

**Supplementary data 3: Non-compliance with protocol**

*Pacemaker not implanted (1 patient)*

- Patient **n°234** had a pre-existing RBBB, and developed a paroxysmal AVB during the procedure. Despite monitoring, no permanent pacemaker was implanted and the patient was discharged. On day 6, he experienced dyspnea during dialysis. ECG revealed 3-AVB, leading to PPI.

*Wrong orientation in GCW (7 patients)*

- Patient **n°9** developed a new onset LBBB associated with 1-AVB (PR 300 ms) but was not monitored with telemetry. He experienced syncope in GCW on day 3, leading to PPI.
- Patient **n°10** had a pre-existing RBBB but was transferred to GCW without any complication.
- Patient n**°68** developed a new onset LBBB but was transferred to GCW without any complication.
- Patient **n°73, 121** had new onset 1-AVB but were transferred to GCW in non-compliance with the protocol, without any complication.
- Patient **n°59, 95** had worsening > 20ms 1-AVB but were transferred to GCW in non-compliance with the protocol, without any complication.

**Supplementary data 4: ECG findings for patients who experienced CD events**

**Patient 1**

ECG baseline

*
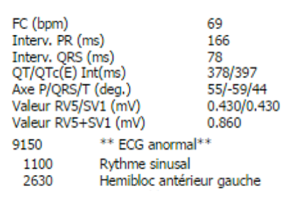
*
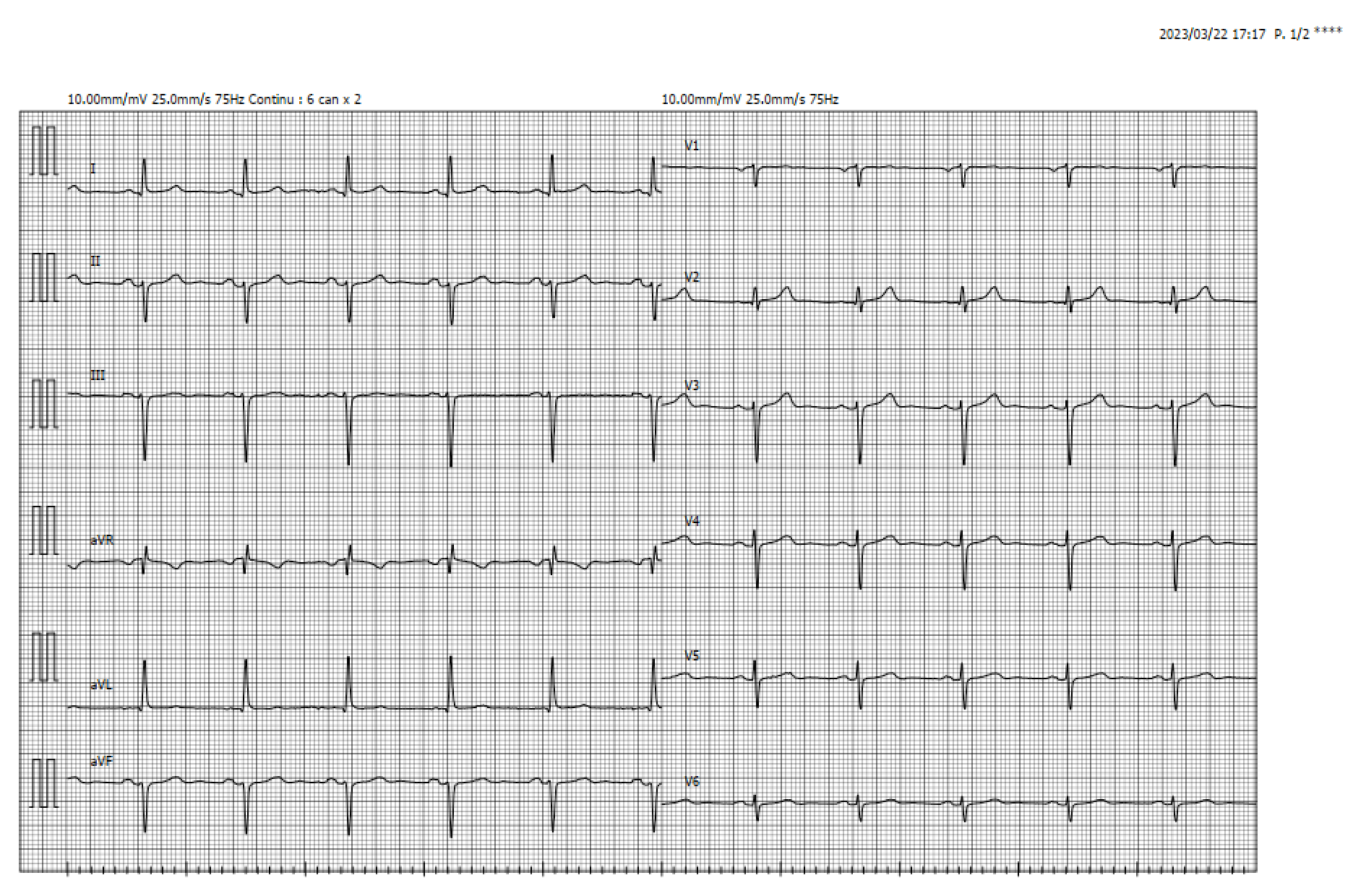


ECG day 3


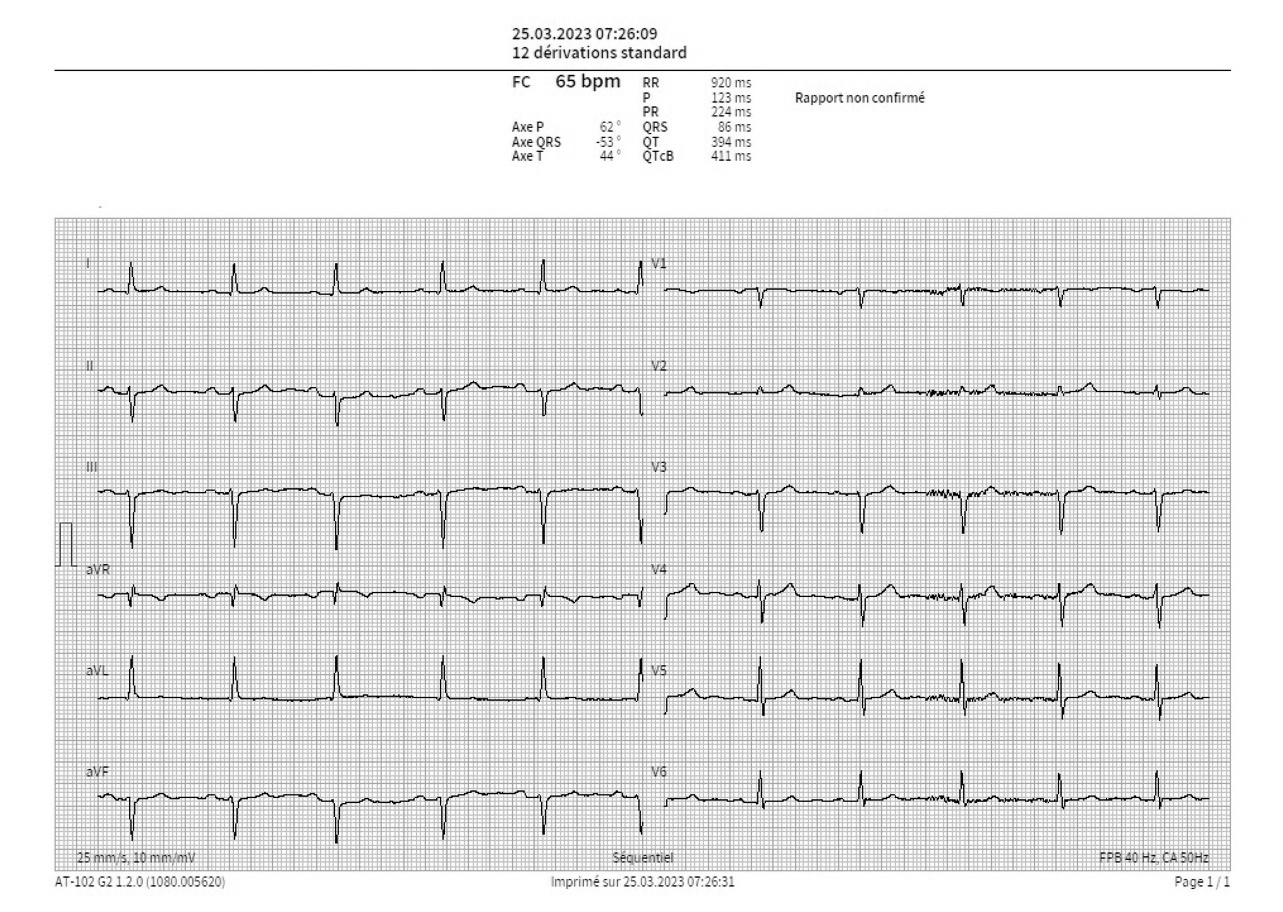


**Patient 2**

ECG baseline


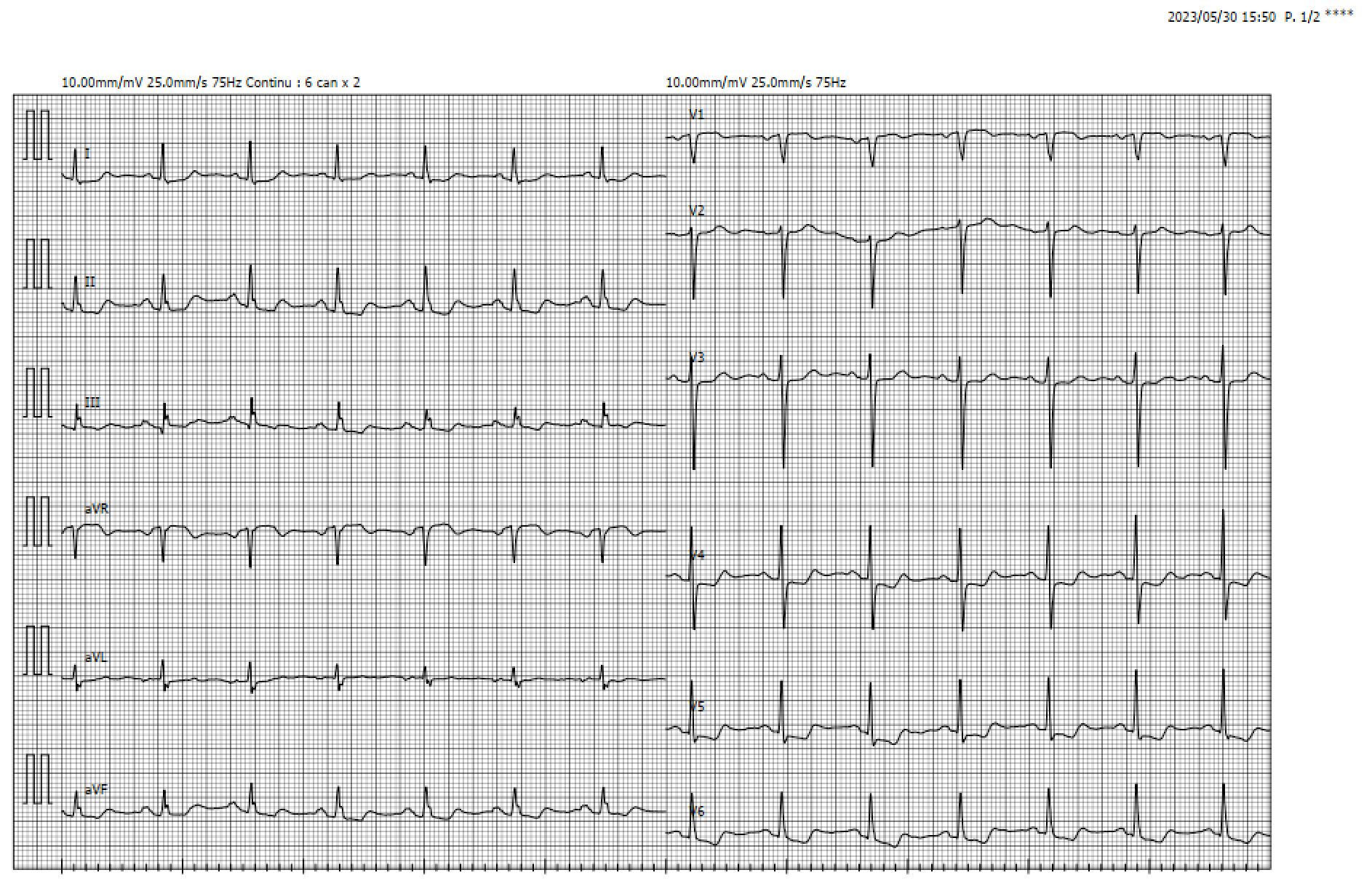


ECG day 6 (discharge)


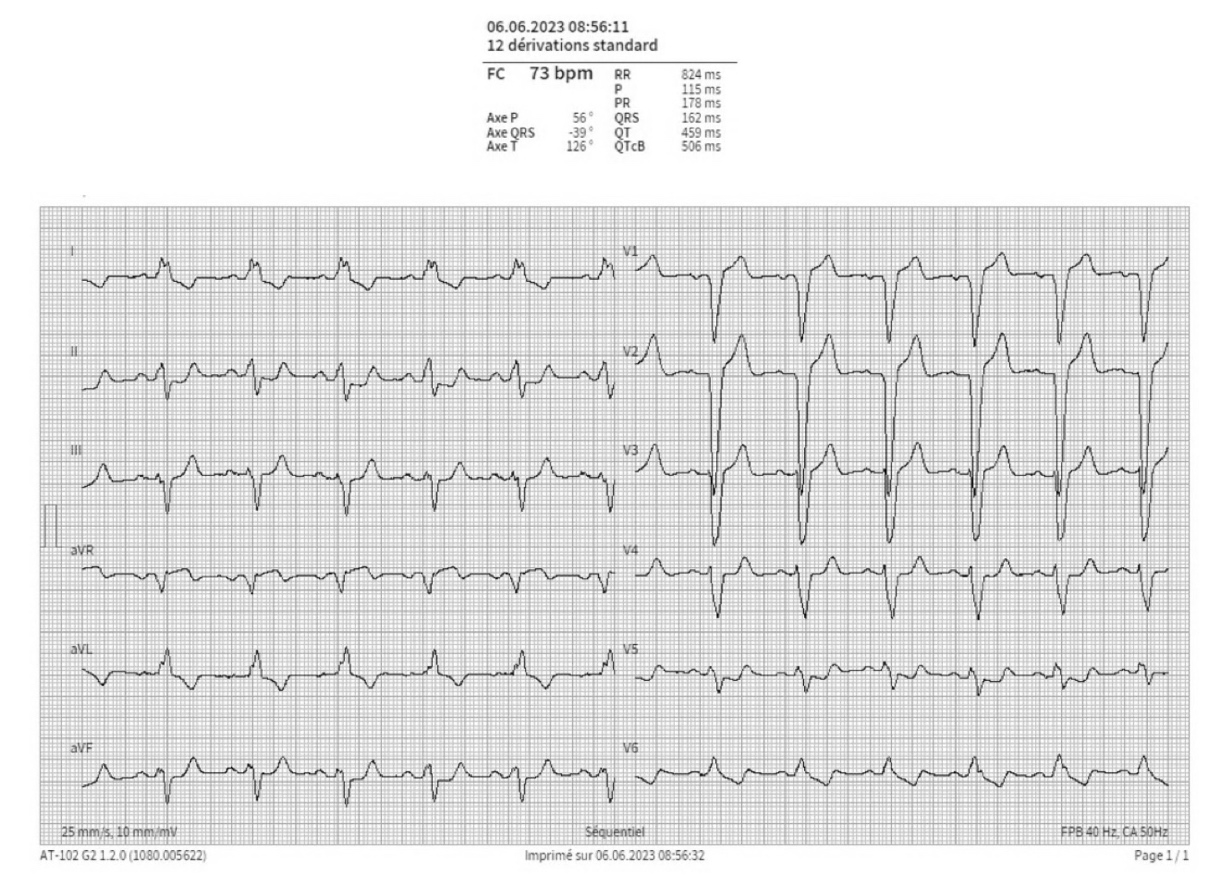


**Patient 3**

ECG baseline


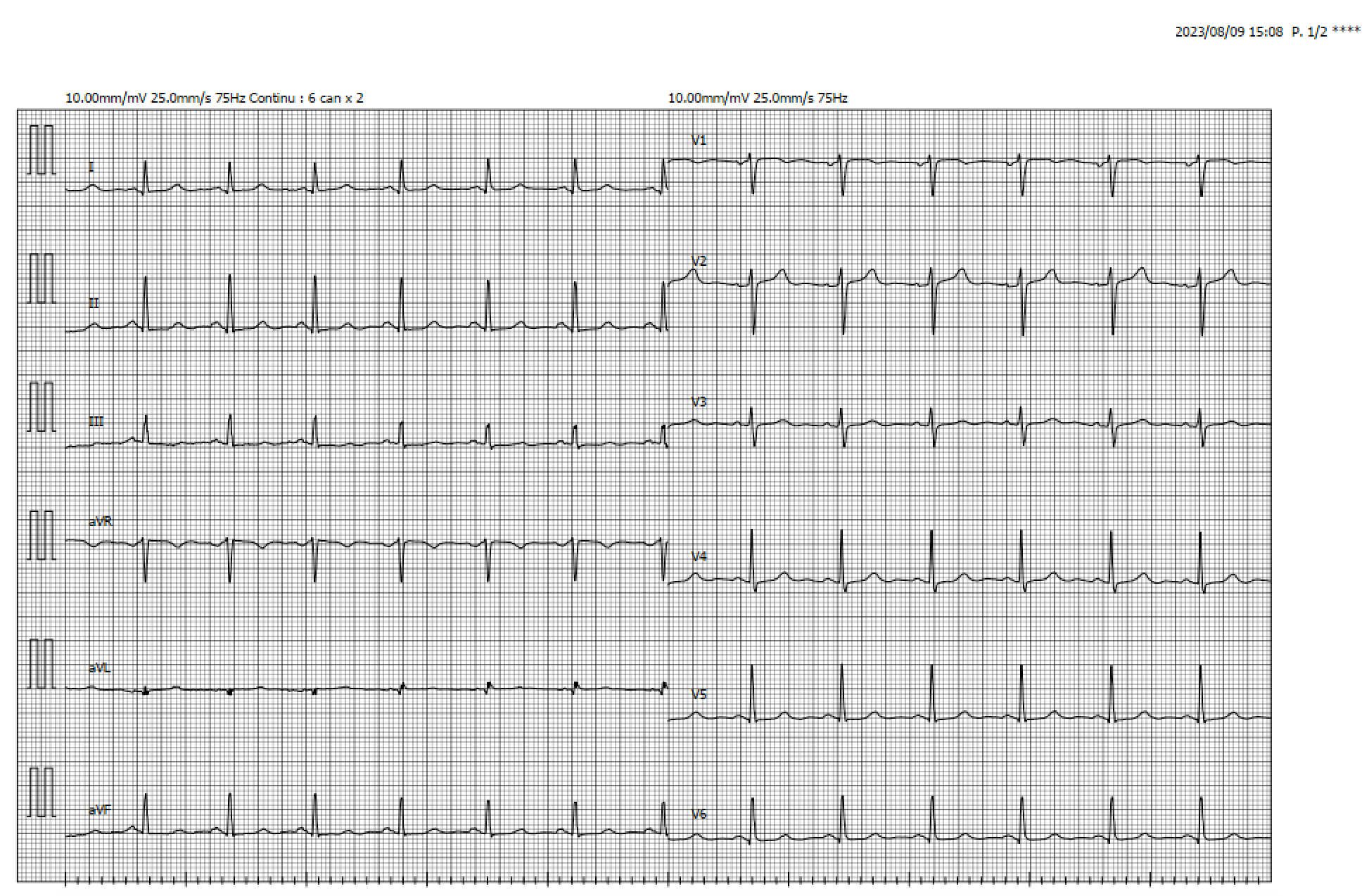


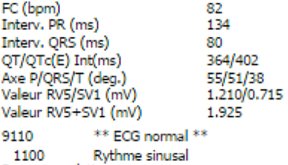


ECG day 3 (discharge)


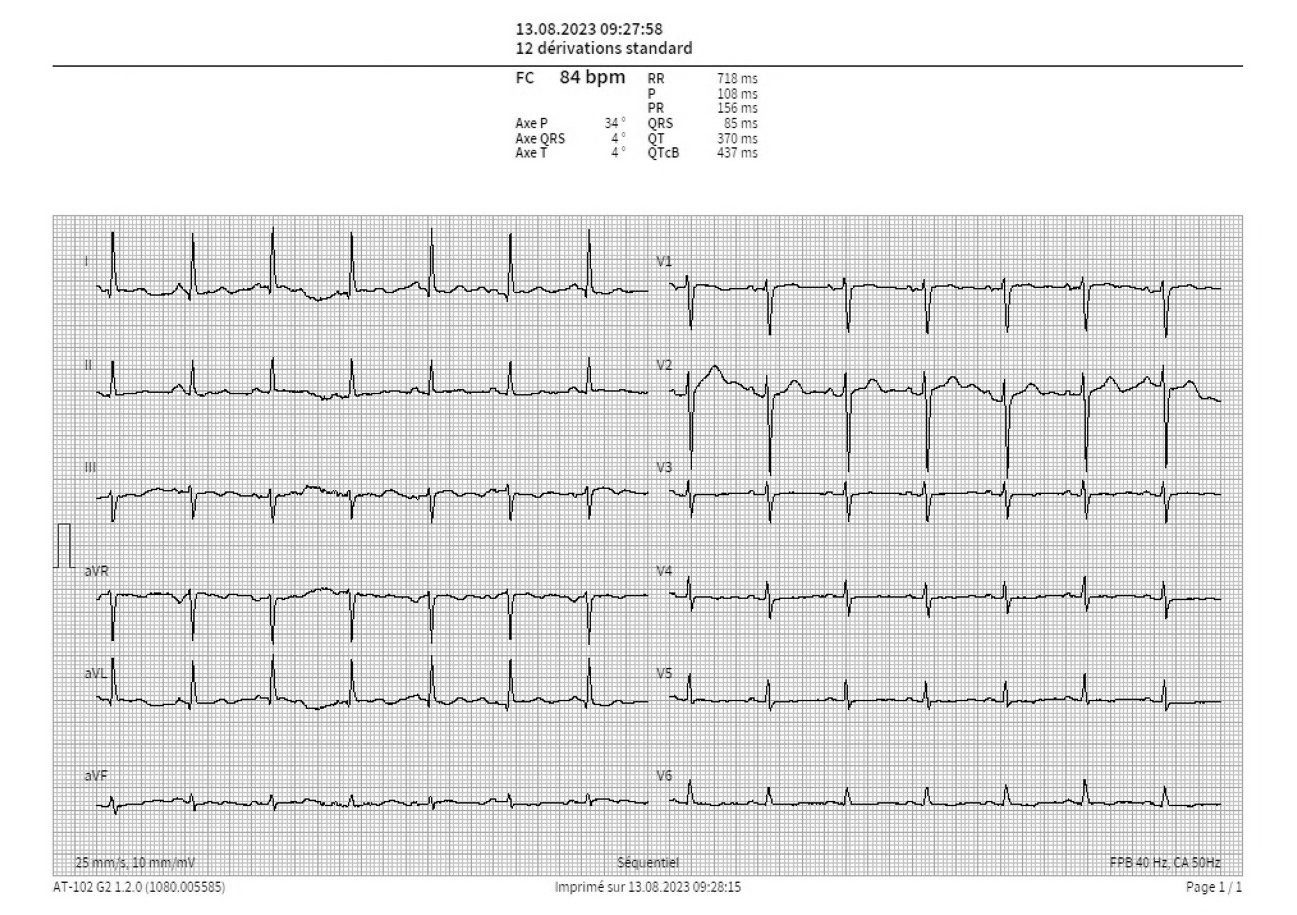


**Supplementary data 5: Permanent Pacemaker rate according to bioprosthesis model**

| **Variable** | **Population** | **No  N = 208** | **Yes N = 42** | **p-value** |
| --- | --- | --- | --- | --- |
| **Bioprosthesis, n (%)** |  |  |  | 0.425^1^ |
| Edward Sapien 3 Ultra | 151 (60.40) | 124 (82.12) | 27 (17.88) |  |
| Edward Sapien 3 | 39 (15.60) | 34 (87.18) | 5 (12.82) |  |
| Evolut Pro+ | 40 (16.00) | 36 (90.00) | 4 (10.00) |  |
| Evolut R | 6 (2.40) | 4 (66.67) | 2 (33.33) |  |
| Navitor | 14 (5.60) | 10 (70.43) | 4 (28.57) |  |
| ^1^Fisher's Exact Test for Count Data with simulated p-value (based on 2000 replicates) | | | | |
